# Improving HIV testing, linkage, and retention in care among South African men through U = U messaging: A study protocol for two sequential hybrid type 1 effectiveness-implementation randomized controlled trials

**DOI:** 10.1371/journal.pone.0309905

**Published:** 2024-11-25

**Authors:** Andrew Medina-Marino, Nkosiyapha Sibanda, Mary Putt, Dvora Joseph Davey, Phillip Smith, Harsha Thirumurthy, Linda-Gail Bekker, Alison Buttenheim

**Affiliations:** 1 Desmond Tutu HIV Centre, University of Cape Town, Cape Town, South Africa; 2 Desmond Tutu Health Foundation, Cape Town, South Africa; 3 Department of Psychiatry, Perelman School of Medicine, University of Pennsylvania, Philadelphia, Pennsylvania, United States of America; 4 Department of Biostatistics, Epidemiology and Informatics, Perelman School of Medicine, University of Pennsylvania, Philadelphia, Pennsylvania, United States of America; 5 Department of Epidemiology, Fielding School of Public Health, University of California, Los Angeles, Los Angeles, CA, United States of America; 6 Division of Infectious Diseases, David Geffen School of Medicine, University of California, Los Angeles, Los Angeles, California, United States of America; 7 Division of Epidemiology & Biostatistics, School of Public Health and Family Medicine, University of Cape Town, Cape Town, South Africa; 8 Department of Medical Ethics and Health Policy, Perelman School of Medicine, University of Pennsylvania, Philadelphia, PA, United States of America; 9 Center for Health Incentives and Behavioral Economics, University of Pennsylvania, Philadelphia, PA, United States of America; 10 Department of Family and Community Health, School of Nursing, University of Pennsylvania, Philadelphia PA, United States of America; Public Library of Science, UNITED KINGDOM OF GREAT BRITAIN AND NORTHERN IRELAND

## Abstract

**Background:**

Increasing HIV testing and treatment coverage among people living with HIV (PLHIV) is essential for achieving global HIV epidemic control. However, compared to women, cis-gender heterosexual men living with HIV are significantly less likely to know their HIV status, initiate anti-retroviral therapy (ART) and achieve viral suppression. This is particularly true in South Africa, where men are also at increased risk of mortality resulting from AIDS-related illnesses. While there is growing knowledge of Treatment as Prevention or the concept Undetectable = Untransmittable (U = U) among PLHIV in Western and high-income countries, the reach and penetration of the U = U message in sub-Saharan Africa remains limited, and few studies have evaluated the impact of accessible and relatable U = U messages on ART initiation and adherence. To address these gaps, rigorous evaluations of interventions that incorporate U = U messages are needed, especially among men in high prevalence settings.

**Methods:**

Building on our U = U messages that we previously developed for men using behavioral economics insights and a human-centered design, we will conduct two sequential hybrid type 1 effectiveness-implementation trials to evaluate the impact of U = U messages on men’s uptake of community-based HIV testing and ART initiation (Trial 1), and retention in care and achievement of viral suppression (Trial 2). For trial 1, a cluster randomized trial will be implemented with HIV testing service site-days (each day at one testing site) randomized to U = U or standard-of-care (SoC) messages inviting men to test for HIV. For trial 2, an individual-level randomized control trial will be implemented, with men initiating ART at six government clinics randomized to receive U = U counselling or SoC treatment adherence messaging. We will incorporate a multi-method evaluation to inform future implementation of U = U messaging interventions. The study will be conducted in the Buffalo City Metro Health District of the Eastern Cape Province and in the Cape Town Metro Health District in the Western Cape Province in South Africa.

**Discussion:**

These trials are the first to rigorously evaluate the impact of U = U messaging on HIV testing uptake, ART initiation and achievement of viral suppression among African men. If effective, these messaging interventions can shape global HIV testing, treatment and adherence counselling guidelines and practices.

## Background

Increasing HIV testing and treatment coverage among people living with HIV (PLHIV) is essential for achieving global HIV epidemic control [1]. Compared to women, cis-gender heterosexual men living with HIV (MLHIV) are significantly less likely to know their HIV status, initiate anti-retroviral therapy (ART) or achieve viral suppression [2, 3]. Moreover, new HIV infections among women are driven, in part, by gaps in testing and treatment among men [4–6]. In South Africa specifically, 79% of women vs. 55% of men reported that they had accessed HIV testing services (**HTS**) in the past 12 months; [7] gaps were even wider for accessing care in rural KwaZulu- Natal [8, 9] Along the continuum of care for HIV-positive individuals in South Africa, women demonstrate higher engagement compared to men [10]. South African men who do initiate ART present with more progressed disease and lower CD4 counts than women, have higher rates of loss-to-follow up (**LTFU**), and are at increased risk for not re-initiating treatment after disengagement from care [9, 11, 12]. As demonstrated in multiple studies across different regions of South Africa, these gaps in care seeking and engagement have led to higher mortality rates for MLHIV compared to women, both prior to ART initiation and while on ART [8, 10, 13, 14]. Ultimately, increasing men’s uptake of HIV testing and treatment initiation must be prioritized to both achieve viral suppression and improve HIV-related health outcomes among men, and to accelerate the decline in HIV incidence among women.

Following landmark studies showing the benefits of Treatment as Prevention (TasP), the Undetectable Equals Untransmittable (U = U) Campaign was launched in 2016 to disseminate scientific evidence that PLHIV who take ART and have an undetectable viral load (<200 copies/mL) cannot sexually transmit HIV [15–20]. This has been further borne out by the recent review which confirms that sexual transmission of HIV is unlikely from an individual whose HIV viral load is <1000 copies /ml [21, 22]. Informed by behavioral economics principles [23–26] and using a human-centered design process [27–31], our team developed U = U messaging for men and conducted a pilot randomized trial of these peer-delivered messages on HIV testing uptake and HIV positive yield among men in Cape Town, South Africa [32]. The U = U messages sought to reduce fear of testing HIV-positive by emphasizing the various health benefits of ART, including the ability to protect sex partners even during condomless sex. The pilot data showed that when presented as part of community-based HIV testing services (HTS), peer-delivered U = U messaging increased the odds of HIV testing uptake by 60% compared to standard messaging about the availability of free HIV testing [32]. We subsequently conducted participatory prototyping to further refine U = U messages to improve ART initiation and early retention in care.

Particularly for men, the U = U message can accelerate progress towards the UNAIDS 95-95-95 targets by reducing anxiety associated with HIV testing (1st 95), encouraging ART initiation (2nd 95), and reducing fear of transmitting HIV to sexual partners by promoting treatment adherence to achieve viral suppression (3rd 95) [33, 34]. While there is growing knowledge of TasP/U = U among PLHIV in Western and high-income countries, the reach and penetration of the U = U message in high prevalent countries has been limited [35–47] and few studies have evaluated the impact of accessible and relatable U = U messages on HIV testing and ART initiation and adherence in the region [48–50]. To address these gaps, rigorous evaluations of interventions that incorporate U = U messages are needed, especially among cis-gendered heterosexual men in high prevalence settings.

## Study aims

Building upon this work, we will conduct two sequential hybrid type 1 effectiveness-implementation randomized trials [51, 52] to compare the impact of U = U messaging versus standard of care on men’s uptake of testing, initiation of ART, and achievement of viral suppression. Our study aims to 1) evaluate the impact of U = U messaging on men’s uptake of HTS and ART initiation; 2) evaluate the impact of U = U messaging on retention in care and viral suppression among MLHIV who initiate ART; and 3) conduct a multi-method evaluation to inform future implementation of U = U messaging interventions. We hypothesize that, compared to standard of care messaging and counselling, U = U messaging will increase HTS uptake by men, increase the number of men initiating ART at 30 days, increase viral suppression at 6 months, and increase retention in care at 12 months. We will also characterize intervention implementation outcomes and identify key, multi-level contextual factors that may block or facilitate implementation of a U = U intervention within HIV service delivery.

## Methods

### Study setting

**Fig 1:**
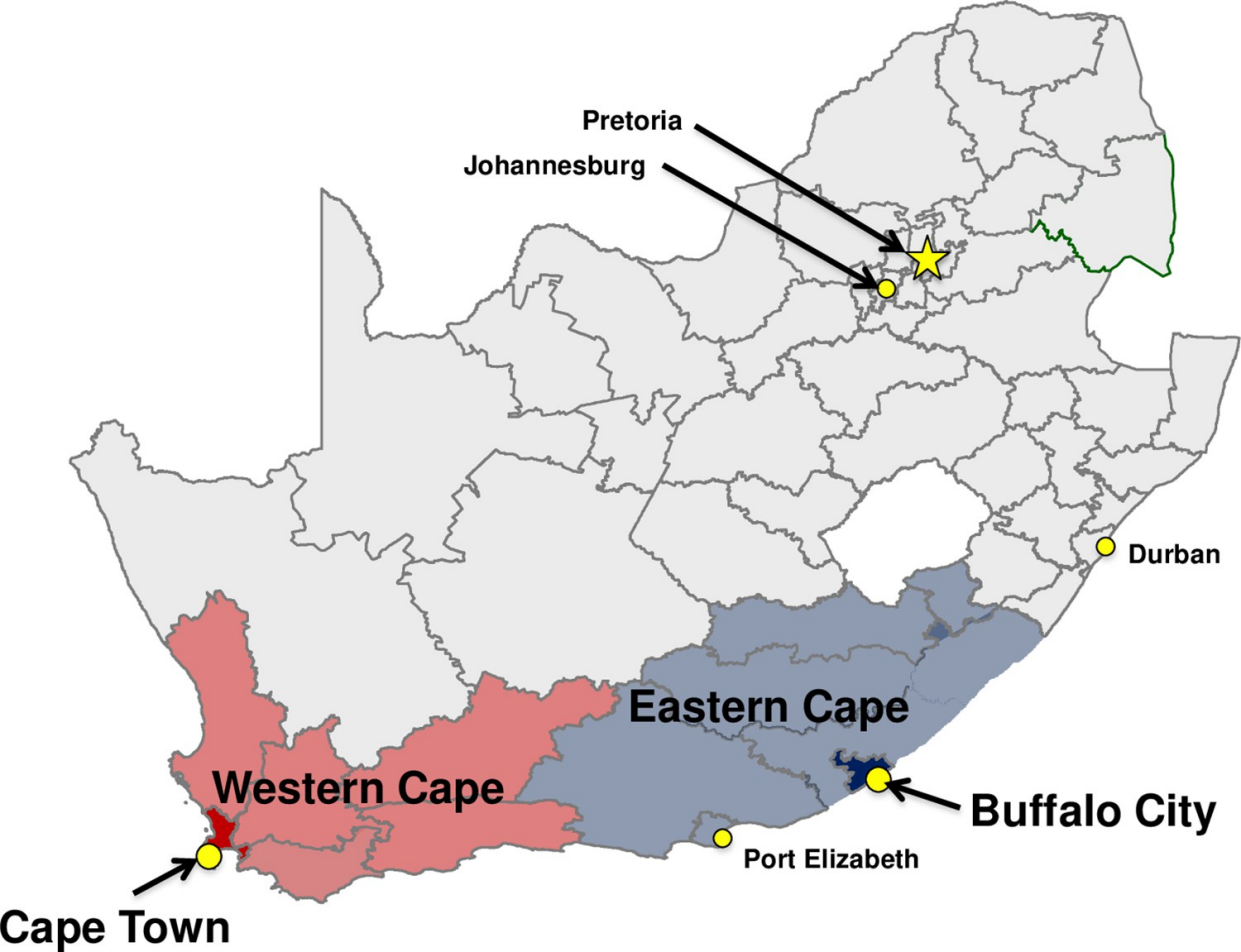
Map of study Districts: Cape Town Metro (Western Cape Province) and Buffalo City Metro (Eastern Cape Province).

We will conduct the study in the Cape Town Metro (CPT; Western Cape Province) and the Buffalo City Metro (BCM; Eastern Cape Province) health districts, South Africa (Fig 1)–two settings with a high HIV prevalence and where our team has existing strong community engagement and health facility partnerships. Of note, the Eastern Cape Province is a historically under-resourced and under-researched province.

In 2022, the estimated adult HIV prevalence in the Eastern Cape Province was 19.4% and in the Western Cape Provinces it was 11.2% [53]. That same year, an estimated 92.6% of individuals living with HIV in the Eastern Cape knew their status, 74.9% were on ART, but only 62.6% were virally suppressed (i.e., <1000c/ml) [54]. In comparison, in the Western Cape, an estimated 93.7% of PLHIV knew their status, 70.3% were on ART, but only 62.4% were virally suppressed (i.e., <1000c/ml) [53, 54]. These statistics are well below the UNAIDS global target for viral suppression of 95%by the year 2025.

Using aggregate South African National Department of Health clinic-level HIV programmatic data, we identified three clinic catchment areas per study district (N = 6) with high HIV prevalence. Study participants for Aim 1 will be recruited from communities within the catchment areas of six large government HIV treatment clinics located in the Kilpfontein and Gugulethu communities of Cape Town (*n* = 3), and the Mdantsane, Duncan Village and Dimbaza communities of Buffalo City Metro (*n* = 3). Study participants for Aim 2 will be recruited from within these same six ART clinics. Residents from these communities are primarily black and Xhosa-speaking. Black South Africans living in peri-urban settings (e.g., Kilpfontein, Gugulethu, Mdantsane and Duncan Village) have among the highest rates of HIV infection in South Africa, while those living in rural settings (e.g., Dimbaza) have among the poorest access to health clinics. Our peri-urban selected communities are densely populated, with a high number of residents living in informal housing; Gugulethu is one of the oldest but fastest-growing township communities in Cape Town, while Mdantsane is one of the largest townships in the country. For Aim 3, we will gather quantitative implementation process measures from recruitment and refusal logs, participant sociodemographic data, tracking tools, and clinic and testing site characteristics, as well as using validated tools during implementation of Trial 1 and 2. We will also conduct qualitative interviews with key stakeholders to inform targets and mechanisms for implementation strategies. Recruitment for Aim 3 will occur through long-standing research collaborations and strong networks and relationships in the region; eligible participants will be sought through meeting announcements, flyers, and email outreach.

### Ethics approval and consent to participate

The protocol, informed consent form and all translations, participant education and recruitment materials have been approved by the ethical review bodies responsible for oversight of research and have been reviewed and approved by the University of Cape Town, Research Ethics Committee (Approval Number: 556/2021) with respect to scientific content and compliance with applicable research and human subjects’ regulations. IRB approvals have also been granted by University of Pennsylvania via IRB reliance on the University of Cape Town. Eastern Cape Provincial Department of Health approval (Approval Number: EC_202204_002) has been obtained to conduct this study in Buffalo City Metropolitan Health District. Western Cape Provincial Department of Health approval (Approval Number: 202209_022) has been obtained to conduct this study in Cape Town. Subsequent to initial review and approval, the responsible Ethics Committees will review the protocol for renewal once every 12 months. The protocol, informed consent forms and all participant materials, and amendments thereof were or will be reviewed and approved by the ethical review bodies responsible for oversight of research. Informed consent will be obtained from all the participants prior to being enrolled in the study. For participants below age 16 years Ministerial waiver of parent/guardian informed consent was reviewed and approved by the University of Cape Town, Research Ethics Committee. The Investigators will submit safety and progress reports to the Ethics Committees at least annually, and within 3 months of study termination or completion. These reports will include the total number of participants enrolled in the study, the number of participants who completed the study, all changes in the research activity, and all unanticipated problems involving risks to human subjects or others.

#### Trial 1 (T1): Uptake of HIV testing & linkage-to-care

**Fig 2:**
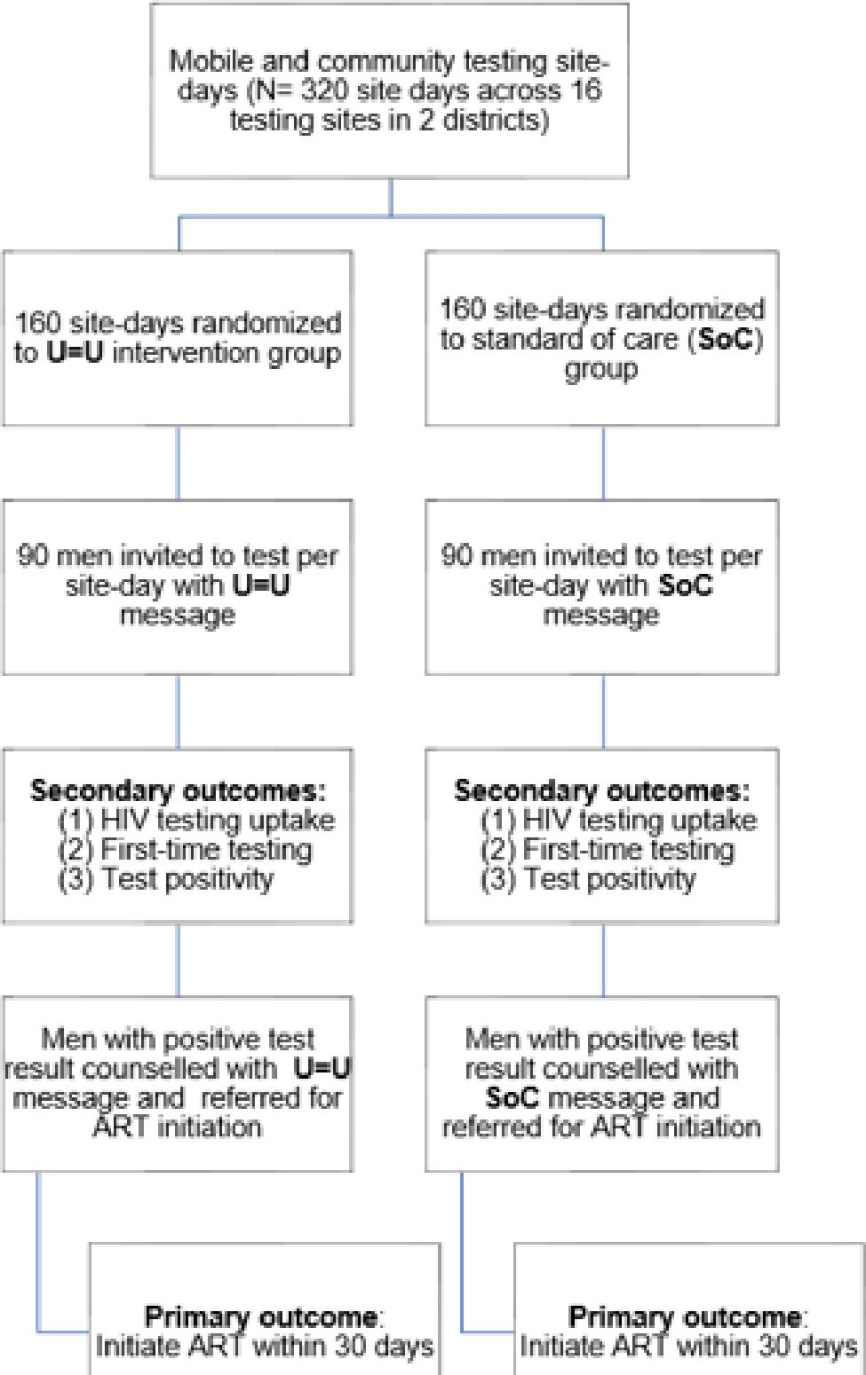
Aim 1 trial design and flow.

*T1 overview and study flow*. To evaluate the impact of U = U messaging on men’s uptake of HIV and ART initiation, we will leverage our well-established mobile HIV testing vehicles (i.e., mobile Tutu Tester Trucks) and pop-up tents in the study communities [55–60]. We will conduct a hybrid type 1 effectiveness-implementation randomized controlled trial, with randomization at the ‘site-day’ level (Fig 2) [61]. A site-day is defined as one day at one testing site. HTS site-days (N = 320) will be randomized to U = U or Standard-of-Care (SoC) messaging to invite men (N = 28,880) to test for HIV. This study design allows us to evaluate the impact of U = U messaging on HIV testing uptake and on ART initiation.

*T1 cluster randomization and placement of testing services*. Based on the clinic catchment area, and in consultation with local community leaders, mobile testing units or temporary testing tents (collectively referred to as community-based HIV testing services; CB-HTS) will be located at high foot-traffic sites (e.g., taxi ranks, entrance/exits to markets, shopping malls and supermarkets) in each clinic catchment area (i.e.,16 unique testing sites). Each site will be assigned a single intervention (SoC or U = U) on each day that the mobile testing units or temporary testing tents are located there. The sequence of interventions for each site was determined randomly, using a blocking scheme that ensures that the number of SoC and U = U days was balanced within each site every 4 or 6 days. Separate randomization schemes were developed for each site so that no sites consistently received the interventions in the same order. Randomization sequence will be determined prior to study launch and revealed to the CB-HTS team the night before each site visit. Western Cape, Eastern Cape Rural and Eastern Cape Urban each had their own research staff who visited the sites and were thus considered as three separate strata. To minimize the potential for contamination (e.g., an individual receiving an invitation card one day and revisiting the site to test the following day), no site will be visited on consecutive calendar days.

**Fig 3:**
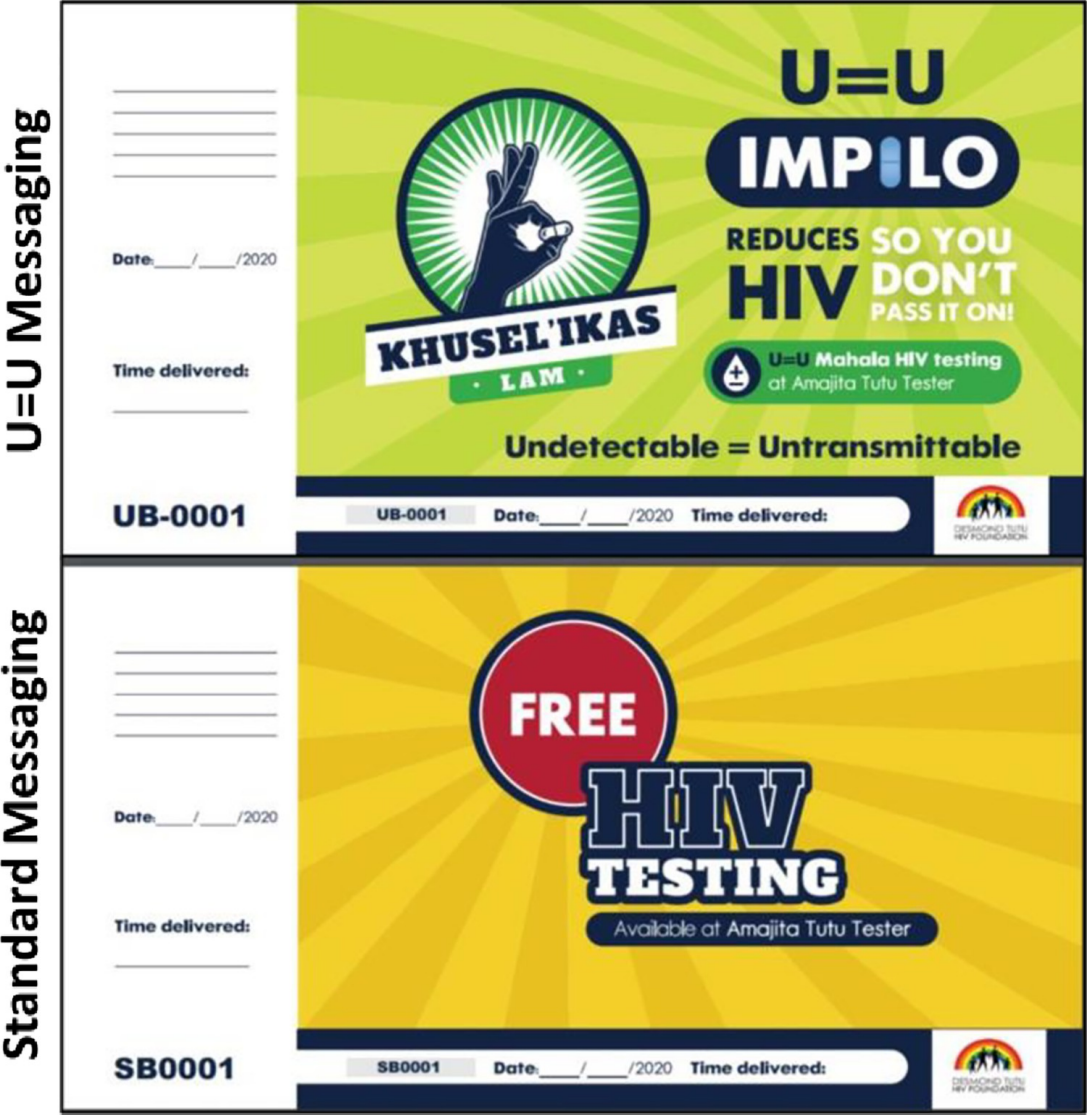
U = U and standard messaging invitation cards for HIV testing services.

*T1 participant recruitment and data acquisition*. Following procedures developed in our pilot trial, [61] trained male peers will approach, invite and provide invitation cards for free HIV testing services (Fig 3) to a total of 28,880 adolescents and men aged ≥15 years who happen to be in the immediate vicinity (±200m) of testing sites. Male peers will deliver the scripted U = U message and U = U invitation card (Fig 3) on intervention days, and the SoC HIV testing message script along with the SoC invitation card on control days; the SoC invitation script is approximately the same length as the U = U script. Invitation cards will be pre-printed with a unique participant identification code (PID). The male peers will write the date, time of distribution and their initials on each invitation card as they are handed out. All men presenting to a HIV testing site will be asked to provide their invitation card to testing site staff. Eligible men presenting a study invitation card for HIV testing will be invited to join the study and asked to provide written informed consent and personal contact details. Regardless of the invitation card a man receives, he will be able to present for testing at any time that day or in the future- i.e., there is no time limitation to when a man receives an invitation card and when he can present for testing.

Using a process successfully implemented during the pilot trial, a site receptionist will record the invitation card’s unique PID, and the date and time of presentation. Reasons for non-enrollment will be recorded. The following inclusion criteria will be applied 1) male; 2) aged ≥15 years; 3) presentation of a study-issued invitation card; and 4) provision of informed consent. Consenting participants will receive a unique study ID number (UID),and be asked to complete a baseline questionnaire administered by study staff prior to being directed to an HIV testing counsellor for a rapid HIV test.

Per South African National HIV testing guidelines, all individuals seeking HTS (i.e., study and non-study participants) will be consented by a certified HIV testing counsellor to receive standard pre-test counselling and an HIV rapid test. Study participants who receive an HIV negative test result, regardless of treatment assignment, will receive standard post-test counselling; and will be offered HIV prevention services (Tutu Testers in Western Cape) or a referral for HIV prevention services at local government clinics (Eastern Cape). Participants in the U = U intervention arm with an HIV-positive test result will receive U = U post-test counselling about ART initiation (Table 1).

**Table 1:** U = U messaging for HIV testing invitation and post-test counseling and ART referral, delivered by male peers (Aim 1).

**HIV Testing Invitation**	Hi! My name is [*peer promoter name]*, and I’m with [*community testing organization]*. Do you know “iMpilo” (*health*, *also sounds like “pill”)*? iMpilo is the latest mahala (*free*) ARV pill that you take once a day if you are infected with HIV. Did you know that iMpilo protects you from getting sick because it reduces HIV in the body—so much so that you can’t infect your partner and family? This is called U = U. It protects you even if you don’t use a condom. Even if you’re drinking. Did you know that? So in no time you’re Ugrand (*strong*, *courageous)* and protecting your partner(s) and family. Your life stays the same. It doesn’t change. You and I can show our kasi (*community)* how to do this thing! You can join in Khusela ikasilam (*protecting our community)*. [*Testing site*] can quickly tell you your HIV status and get you iMpilo for mahala (*for free)*. Take this invitation with you. See you there!
**Post-test counseling/ART referral**	I know you may be feeling sad/overwhelmed/angry about your HIV status and learning you are living with HIV. But there is HOPE and life doesn’t have to change if you take charge today. Learning you have HIV is not like before in our parents day. You (& I—*if counsellor is HIV+ and discloses*) can live a long, normal life like thousands of other South African men living with HIV! I will show you how! Remember hearing about iMpilo before? iMpilo is the latest mahala ARV pill that you take once a day if you are infected with HIV. Now that you know your status, you’ll take iMpilo daily to protect you from getting sick. iMpilo reduces HIV in the body—so much so that you can’t infect your partner and family. This is called U = U. iMpilo can protect you even if you don’t use a condom. Even if you’re drinking. Your life stays exactly the same as before today, it doesn’t change. So in no time you’re Ugrand. By taking iMpilo daily and not missing your iMpilo pickups, you stay healthy and protect your partner(s) and family from getting HIV. You and I can show our kasi how to do this thing. You can join in Khusela ikasilam. How are you feeling now? You can start iMpilo for mahala for free at your preferred, nearest clinic where they will start you on treatment on the same day so you *can* protect your sex partners and family from getting HIV and live a life just like before. Where do you want to start treatment? What is your plan for starting (when will you go and with whom)? Take this referral letter with you. We’ll follow up with you in a few days to find out if you are on your way to reducing HIV in the body with iMpilo. Do you have questions about iMpilo or how to get started?

Participants in the SoC condition who receive an HIV-positive test result will receive standard (guidelines-based) post-test counselling about ART initiation. Community members (male or female) who passively present to any of our testing venues, or men who do not present with an invitation or do not provide consent will receive standard testing per South African national guidelines [62] including standard pre- and post-test counselling.

*T1 outcomes and data analysis plan*. The primary outcome will be the intention to treat, which we defined as ART initiation within 30 days of receiving a positive HIV result. ART initiation will be established through participant self-report (via phone calls) and from the National Health Laboratory Service (NHLS) or clinic records where available. In the event of any discrepancies or disagreement between self-report, lab records, or clinic reviews, priority will be given to the information documented in the lab records to define the primary outcome. NHLS tracks lab results including HIV testing, viral load (VL) and CD4. As a proxy for ART initiation, we are identifying CD4 or viral load results to identify when baseline bloods were taken prior to ART start, or to identify people already on ART (prior VL or CD4). The NHLS laboratory system allows us to see provincial level data. Therefore, by having access to NHLS data, we will be able to track participants regardless of their migration status.

Generalized estimating equations (GEE) with a logistic link function and an independent working correlation structure will be used to estimate the odds of ART initiation following U = U versus SoC messaging and will be averaged across sites. The GEE method takes into account clustering by site. A significance level of 0.05 will be employed and calendar quarter will be controlled for to account for seasonal variability in behaviour.

Secondary outcomes will include HIV testing uptake, first-time testing, and test-positivity among those who receive the invitation cards as well as ART initiation among those who test positive for HIV. For this, mixed effects models will be used to estimate: 1) the difference in HIV testing uptake comparing U = U to SoC messaging exposure, and 2) between-site and within-site HIV-testing uptake variability. By ‘between-site’ variability we mean the random variation in HIV testing uptake among different sites, due to intrinsic differences in the populations at these sites. For example, men who are unemployed may have more time available to test than those who are employed; different rates of employment among men sampled at different sites could thus lead to between-site variation in HIV testing uptake. Variability within-site is a reflection of local conditions at each site. For example, at any site, potential participants might be more likely to take the time to test on a pleasant day than on a rainy day; changing weather is potentially one source of within-site variability.

Similar as for the primary outcome analyses, GEE and working correlation structure will also be used for the secondary outcomes. To better identify where in the HIV testing-linkage to care sequence U = U may specifically have an impact, we will estimate the odds ratio and 95% CI for U = U versus SoC for each subsequent outcome conditional on results for the previous outcome (i.e., the outcome of testing positive given the behaviour of presenting for testing, and the behaviour of ART initiation given the outcome of an HIV positive result). Participant groups will be characterized at each step of the sequence to assess how U = U messaging might be influencing specific groups of individuals to test or link to care. Causal methods (potential outcomes) will be used to assess if individuals who test positive following U = U versus SoC messaging are more likely to initiate ART.

*T1 sample size calculations and statistical power*. A sample size of 28,880 invitation cards distributed (i.e.,14,440 per intervention message) gives us 84% power to detect a risk difference in our primary outcome (yield, or ART initiations as a percent of invitations distributed) of 0.156%, and a risk ratio of 2.57. In other words, we can detect a difference in yield of 0.1% (1 ART initiation per 1000 invitation cards) for SoC versus 0.257% (2–3 ART initiations per 1000 invitation cards) for the U = U intervention. Our sample size calculations are informed by pilot data [61], experience in the field, and recent HIV incidence.

For the U = U intervention arm, we estimated that 15% would present for testing, 3.5% of those who test would test HIV-positive, and 49% of those receiving a positive test result would initiate ART, for a yield of 0.257%. For the SoC arm, we estimated that 10% would present for testing, 2.5% of those who test would test HIV-positive and 40% of those testing positive would initiate ART, for an overall yield of ART initiation among those invited to test of 0.10%. In order to address clustering by site, sample size calculation assumed a within-site-day intraclass correlation (ICC) of 0.01 for participants sampled on the same day at a single site, and a between-site-day ICC of 0.009 for participants sampled on two consecutive sampling days at a site. A discrete-time decay correlation structure using an exponential decay function of the time ‘j’ between site days allowed the correlation between participants sampled at longer intervals apart to decrease over time. Power calculations were conducted by uploading a design matrix generated in R(V4.2.2) to Shiny CRT [63]. To achieve this sample size, we will distribute 90 invitation cards per site day for a total of 320 site days (160 for U = U intervention and 160 for SoC).

#### Trial 2 (T2): Retention in care and viral suppression

*T2 overview and study flow*. To evaluate the impact of U = U messaging on retention in care and viral load (VL) suppression among HIV-positive men who initiate ART, we will conduct a hybrid type 1 effectiveness-implementation randomized controlled trial. Randomization will be stratified by the 6 study clinics, to account for differences in baseline viral loads of the catchment area populations initiating ART within each clinic. Individual-level randomization will be used with random block sizes of 4 to 8. The study staff counsellors will recruit men who are either newly initiating or re-initiating ART at study clinics in Cape Town (*n* = 3) and Buffalo City Metro (*n* = 3). Inclusion criteria for trial 2 are as follows: 1) male 2) aged ≥15 years 3) newly initiating ART or re-initiating ART after 6 months of being lost-to-care; 4) live in Buffalo City or Cape Town Metro Health Districts; and 5) provision of written informed consent. Participants (N = 1100) will be individually randomized to receive either U = U messaging (intervention group; *n* = 550) or SoC messaging (control group; *n* = 550) as part of ART initiation counselling and during routine HIV care and treatment. Our trial design allows evaluation of both shorter-term impact and longer-term durability of the U = U messaging for ART adherence and treatment retention.

**Fig 4:**
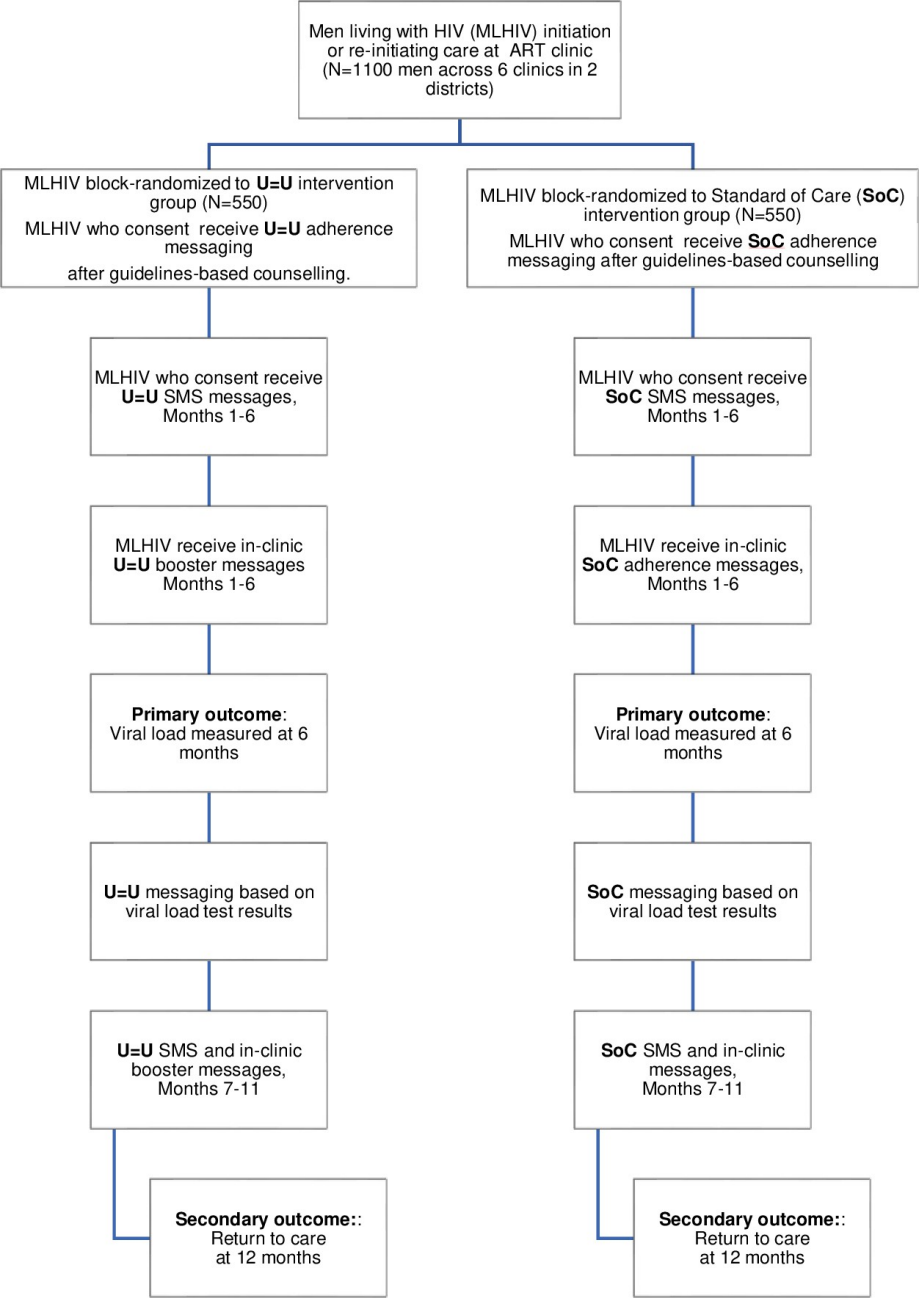
Aim 2 trial design and flow.

*T2 participant recruitment and data acquisition*. All participants will receive standard ART initiation and adherence counselling from a DoH clinic nurse per South African National Guidelines (Fig 4) [62]. Thereafter, study staff counsellors will either (1) deliver U = U initiation messaging, or (2) reinforce SoC messaging through a guidelines-based script of comparable length (Fig 4). Additionally, participants will also receive a small business-sized card either re-emphasizing the U = U or the SoC message [64].

**Fig 5:**
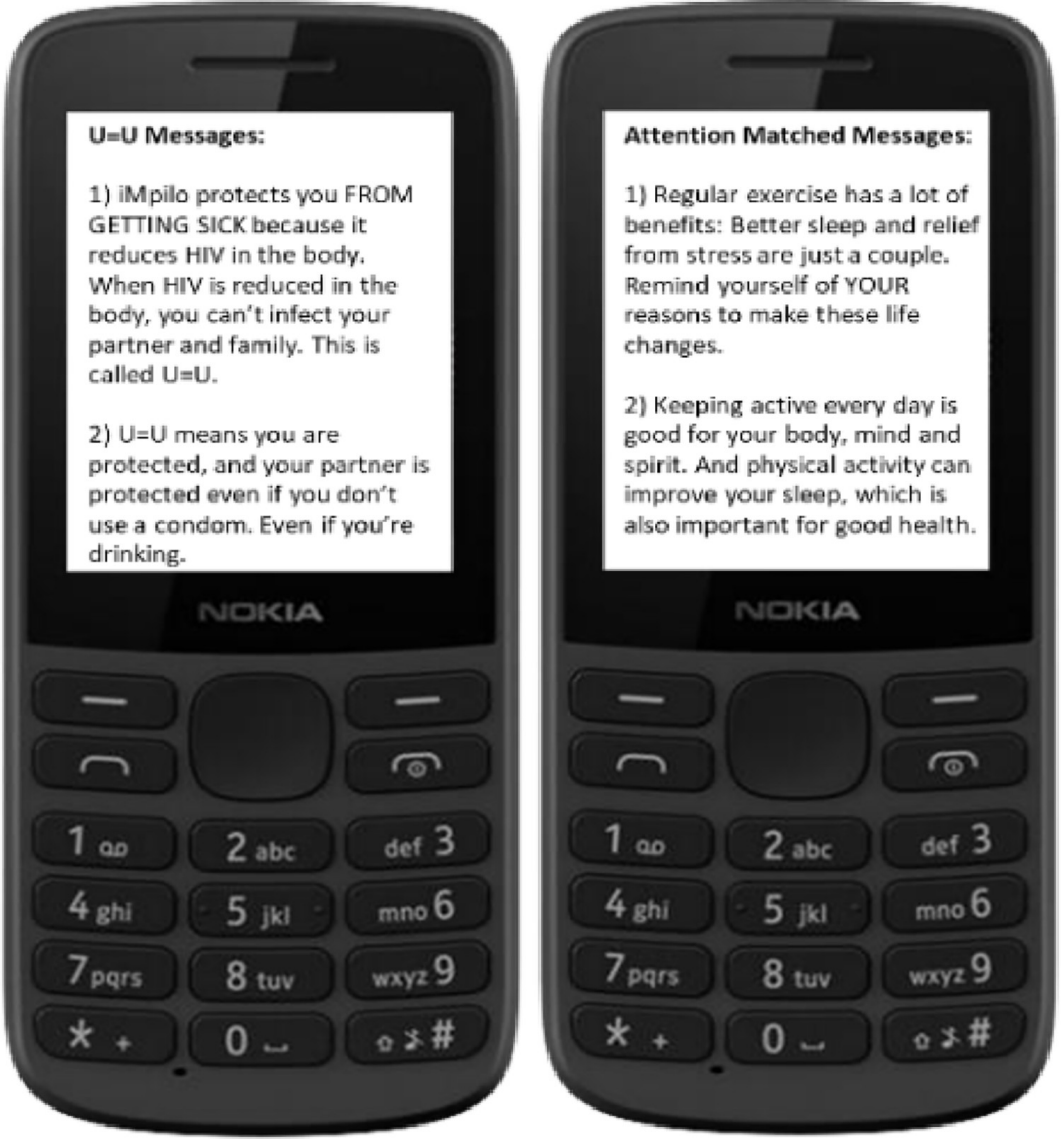
Example U = U and attention matched SMS monthly booster messages.

At enrolment, participants will be informed that they have the option to receive monthly SMS messages (Fig 5). A mid-month U = U or Attention Matched Control one-way text message will be sent to participants opting to receive SMS messages (Fig 5). Monthly text messaging is not part of the SOC protocol in South Africa, thus our decision to employ an attention matched control message for the SoC participant group. SMS paradata will be collected to track message delivery and receipt.

Study staff counsellors will in addition verbally deliver U = U or reinforce SoC adherence messaging (Table 2) to participants during routine medication refill visits, which are scheduled monthly. Paradata from SMS messages and number of clinic visits attended will be recorded to enable mediation analysis [65, 66]. In accordance with South African ART clinical and adherence guidelines [62, 67], clinic nurses provide patients on ART with feedback based on VL test results. Study participants in the U = U condition with a suppressed VL (<50 copies/mL) will receive Basic U = U messaging, while those with an unsuppressed VL (≥50 copies/mL) will receive Enhanced U = U messaging (Table 2). Participants allocated to SoC will receive guidelines-based reinforced SoC adherence messaging based on their VL [68].

**Table 2:** U = U messaging for ART initiation, monthly SMS messaging, in-clinic booster messaging, and viral load results counselling (Aim 2).

**ART (re-)initiation Adherence Messaging**	I know you may be feeling sad/overwhelmed/angry about your HIV status and learning you are living with HIV, but there is HOPE and life doesn’t have to change if you take charge today. Learning you have HIV is not like before in our parents’ day. You (& I—*if counsellor is HIV+ and disclosed*) can live a long, normal life like thousands of other South African men living with HIV! I will show you how! Do you know iMpilo *(health*, *also sounds like “pill”*)? iMpilo is the latest mahala *(free)* ARV pill that you will take once a day if you are infected with HIV. Taking iMpilo daily will protect you from getting sick because it reduces HIV in the body—so much so that you can’t infect your partner and family. This is called U = U. iMpilo will protect you even if you don’t use a condom. Even if you’re drinking. Your life stays exactly the same as before today. It doesn’t change. So in no time you’re Ugrand *(strong*, *courageous)*. By taking iMpilo daily and not missing your iMpilo pickups, you stay healthy and protect your partner(s) and family from getting HIV. You and I can show our kasi *(community)* how to do this thing. You can join in Khusela ikasilam *(protecting the community)* How are you feeling now? You will start (iMpilo/ART) for mahala today, so you can protect your sex partners and family from getting HIV and live a life like before I’ll show you where to go now to get your first iMpilo. It’s important to come back for all your appointments so you can keep getting iMpilo. In six months, we’ll do a test to show you that iMpilo is reducing HIV in your blood. You’ll see! Do you have questions about iMpilo or getting started?
**In-Clinic Adherence Booster**	It’s nice to see you again! How have you been? How is your time with iMpilo going? Remember that life doesn’t have to change while you are on treatment. Having HIV is not like before in our Parents’ day. You (& I—*if counsellor is HIV+ and disclosed*) can live a long, normal life like thousands of other South African men living with HIV! I hope you’re finding that iMpilo is easy and modern. Remember that taking iMpilo daily protects you from getting sick because it reduces HIV in the body—so much so that you can’t infect your partner and family. Remember that this is called U = U. iMpilo is protecting you even if you don’t use a condom. Even if you’re drinking. I hope you’re feeling Ugrand! By taking iMpilo daily and not missing your iMpilo pickups, you are staying healthy and protecting your partner(s) and family from getting HIV. You and I can show our kasi how to do this thing! You are Khusela ikasilam *(protecting the community*). It’s important that you keep coming back for all your appointments so you can keep getting iMpilo. In six months, we’ll do a test of your viral load to see if the treatment is working. You’ll see! Do you have questions about iMpilo?
**Viral Load Test Result Report-Back Messaging**	**Basic message (viral load <50 copies/mL):** We checked your blood to see if the ARVs are working. Good news! The result is that iMpilo has reduced HIV in your blood–so low that we can’t even see it. Remember how we talked about iMpilo before? With iMpilo you have reduced HIV in the body—so much so that can’t infect your partner and family. This is called U = U. It’s great that you have reached U = U. Remember that iMpilo can protect you even if you don’t use a condom. Even if you’re drinking. Your life can stay exactly the same as before. You should feel Ugrand *(strong*, *courageous)*—by taking iMpilo daily and coming to your appointments you are staying healthy and protecting your partner(s) and family from getting HIV. You are showing your kasi how to do this thing! You are Khusela ikasilam *(protecting the community)*! Let’s talk about how to keep up with iMpilo so it can reduce this HIV in your blood, protect your family, and keep you on the road to health. **Enhanced message (viral load ≥50 copies/mL):** We checked your blood to see if the HIV Is reduced in your body from taking iMpilo. The result is that we are still seeing HIV: your viral load is [*value*] and we want to help you get it down so low that our test can’t even see it. Remember how we talked about iMpilo before? When you take iMpilo every day, it reduces HIV in the body—so much so that can’t infect your partner and family. This is called U = U. We want to help you reach U = U. Remember that iMpilo can protect you even if you don’t use a condom. Even if you’re drinking. Your life stays exactly the same as before. Would you like to be Ugrand *(strong*, *courageous*) by taking iMpilo daily and coming to your appointments so that you can stay healthy and protect your partner(s) and family from getting HIV if they are not living with HIV? Would you like to show your kasi (*community*) how to do this thing? Would you like to Khusela ikasilam *(protect the community)*? How are you feeling now? Let’s talk about why it might be hard for you to come to appointments or to take your iMpilo every day. Let’s talk about how we can help you get to U = U.

*T2 outcomes and data analysis plan*. The primary outcome will be the proportion of MLHIV who are virally suppressed at 6 months (impact) and will be analysed using logistic regression with clinic fixed effects. The secondary outcome will be the proportion of MLHIV retained care at 12 months (durability) and will be analysed using logistic regression with clinic fixed effects with outcomes as (1) the number of clinic visits attended prior to six months, and (2) presentation for VL testing at 12 months. VL testing is conducted at 6 months, 12 months and annually thereafter by the Department of Health in accordance with South African national HIV care and treatment guidelines. Should a VL not be recorded in a participant’s medical record, we will consult the SA-NHLS laboratory information system to determine if and when VL testing was performed and record the participant’s VL should it be available. At the 6- and 12-month mark, each participant’s clinic-based medical records will be abstracted for: 1) ART refill and clinic attendance history, and 2) VL results.

All participants lost to follow-up at 6 months will be contacted by study staff using the contact information provided at study enrolment to assess self-reported treatment engagement and invited to return to the clinic to complete a study questionnaire. At 12 months, all participants will also be asked to complete an RedCap administered endline questionnaire in the same manner as the baseline questionnaire. A causal mediation analysis will be conducted to explore direct and indirect effects of the U = U intervention on VL suppression with number of visits as the mediating variable. Pre-specified subgroups for the analyses of the primary and secondary outcomes (the proportion of MLHIV retained in care at 12 months) are those participants: (1) initiating ART for the first time vs. re-initiating ART and (2) Eastern Cape versus Western Cape. For secondary analyses the primary analysis will be repeated using as outcomes:

*Sample size calculations and statistical power*. Trial 2 is an individually randomized trial. A sample size of 1100 is powered to detect an 8% difference in the primary outcome. For a two-sided Type I error rate of 0.05, and using a two-sample proportions test as the basis of the calculation, we have 80% power to discern between 70% (U = U intervention) versus 62% (SoC intervention) viral suppression at 6 months.

#### Implementation assessment

*Overview and study flow*. We will use the RE-AIM and Consolidated Framework for Implementation Research (CFIR) frameworks, and informed by behavioural economics [69–74], and behavioral design [75] to identify implementation barriers and facilitators of the U = U interventions through structured surveys and qualitative interviews. Perceptions and intervention mechanisms will be explored to inform how the U = U interventions could be implemented and scaled in the future. RE-AIM will inform the identification, collection, and analysis of quantitative survey indicators related to the U = U interventions over the course of the two sequential trials. CFIR will guide a qualitative exploration of the conditions and contexts shaping successful implementation through interviews with key stakeholders (i.e., participants, research/clinic staff, health department managers, policy makers).

*Participant recruitment and data acquisition*. We will recruit and consent HTS providers (*n* = 20), ART nurses (*n* = 24), clinic operation staff (*n* = 12) and clinic service managers (*n* = 12) representing both the Cape Town and Buffalo City Metro health districts (*n* = 68 total); this sample size is sufficient to generate stakeholder preferences for provide a signal about implementation outcomes within and across study sites and districts for both trials, without putting undue burden on clinic or testing site staff.

*Qualitative interviews*. We will also purposively recruit, consent and interview 40–60 individuals from seven key stakeholder groups. Key stakeholder groups will include: 1) MLHIV who attend the testing sites Including those who test positive but do not initiate (trial 1) or clinics (trial 2) where our trials are conducted 2) health care providers (i.e., nurses or physicians) at the clinics where the retention and viral suppression trial is conducted 3) lay health workers and counsellors 4) clinic leads 5) district, provincial and national departments of health National Department of Health (NDOH) 6) PEPFAR implementing partner leads and 7) South Africa HIV Task Force members. Recruitment will occur through long-standing research collaborations and strong networks and relationships throughout South Africa; eligible participants will be sought through meeting announcements, flyers, and email outreach. The trial 1 interview guide will incorporate results from the Aim 1 trial, presented narratively and graphically. The trial 2 interview guide will incorporate both Aim 1 and Aim 2 trial results, also presented narratively and graphically. Key themes covered in the interview guide will include perceptions of the U = U message, recommendations for implementation of U = U within and South African Department of Health guidelines. Interviews will be conducted by one of the study PI’s or project manager currently pursuing doctoral training.

To describe intervention effectiveness and inform future implementation efforts, we will gather quantitative implementation process measures from recruitment and refusal logs, participant sociodemographic data, tracking tools, and clinic and testing site characteristics. These data will be collected throughout the implementation of trials 1 and 2. To identify key, multi-level contextual factors that may block or facilitate implementation of a U = U intervention within HIV service delivery, we will conduct 1) in-depth qualitative interviews with key stakeholders to inform targets and mechanisms for implementation strategies, and 2) validated quantitative implementation measures. Data will be collected at the mid-point of recruitment for each trial at each site.

*Data analysis plan*. Passively and actively collected process indicators informed by the RE-AIM framework will be synthesized and summarized iteratively over the course of the two trials. Descriptive statistics and comparisons by study site, provider type, and trial will be used to assess implementation success, interpret trial results, and identify implementation targets and mechanisms. Verbatim transcripts of semi-structured interviews will be entered into NVivo qualitative analysis software (QSR international, Burlington MA) for data management and analysis. Two separate qualitative analyses will be conducted. First, a hybrid inductive-deductive thematic analysis [76] will be conducted using both *a priori* codes related to CFIR domains and constructs, as well as *de novo* codes that emerge from the data.

Two team members will code the first three interviews by consensus, then the next three interviews independently and review them together to ensure consensus. Thereafter, all interview transcripts will be coded by two team members independently, with double-coding of 20% of interviews to ensure inter-rater reliability. Outstanding coding questions and disagreements will be resolved by consensus of the team. We will qualitatively analyze themes across interview rounds and stakeholder types to identify key opportunities and challenges for implementation of the U = U interventions. Second, the NUDGE framework [75, 77, 78] will be employed to uncover specific barriers to implementation of evidence-based interventions through the application of behavioral insights to rich contextual data such as in-depth interviews. Behavioral barriers to intervention adoption, adaptation, fidelity, and maintenance identified through this process will inform future implementation strategies and policy recommendations.

## Data management

### Participant identification

All eligible study participants that provide informed consent will be assigned a Participant Identification Number (PIN) by REDCap. This PIN composition will allow us to identify participants at the community and clinic level, thus ensuring convenient integration of various datasets to carry-out various level-based analysis approaches. Participants’ allocated PIN will be used for the duration of the study at each data collection point. Results from other data sources such as the South African electronic TB register (ETR.net) and District Health Information Systems (DHIS), as well as laboratory databases and clinic registers will be back captured onto REDCap, therefore making REDCap the primary data collection and storage platform for this study. A secure link-log (linking participant identifiers such as names, date of births, etc. and the PIN) database will be developed and stored in a separate REDCap database accessible only to the Principal Investigators (PIs) and those study staff granted access upon approval by the PIs.

### Data storage

All study databases will be hosted on secure cloud-based platforms. Datasets will be merged using primary identifiers and stored in a secure, password-protected, web-based database which will only be accessible to authorized project staff. Paper records of participants will be kept in lockable filing cabinets. Paper records, excluding informed consent forms, will only contain PINs. Qualitative data including audio files and password-protected transcripts will be stored on a secure, access-controlled cloud-based database (Sharepoint). As per study protocol, All hard copies of data, electronic files, and databases will be stored securely for up to five years after the completion of the study or as required by the institutional review boards and prevalent South African legislation. This will be communicated to all eligible participants as part of the consenting process.

### Data quality

Data quality will be assured by automated data quality checks and skip patterns in REDCap, field-based data quality clerks and office-based data administrators. Quality checks will be performed daily, with inconsistencies rectified using our data query resolution platform. Scheduled and unscheduled quality inspections will be performed on a randomly selected 10% of participants.

### Data and safety monitoring

All research staff will be trained to identify, probe for and report adverse events (AEs) and social harms (SHs). Occurrence of AEs and SHs, will be collected at every visit. All AEs and SHs reported outside of research visits/activities will also be documented and reported. An AE list will be compiled and reviewed by the Data Safety and Monitoring Board (DSMB) which is set up be set up for this study and has ultimate ability to terminate the trial should interventions prove to have unacceptable risk. All DSMB members will have no direct association with this study or the study sponsors.

## Ethical considerations

### Consenting process

Staff will read all eligible participants a brief description of the study. Interested participants will then be read aloud the IRB-approved study consent form in their preferred language, which will provide specific information about HIV infection, the consequences and treatment of HIV, and study risks and benefits, and then will be invited to participate in the study. Those interested in the study will be consented in a safe, private space in or near the study clinic, enrolled and randomized; reasons for ineligibility or refusal will be recorded.

For adolescent boys 15–17 years old, a Ministerial waiver of parent/guardian permission was reviewed and approved by the University of Cape Town, Research Ethics Committee. This is in line with the South African National Department of Health’s 2015 publication, ‘Ethics in Health Research’, which states that ‘in particular circumstances, e.i. for reasons of sensitivity, like discussion about sexual activities, substance abuse etc., it may be desirable and ethically justifiable for minors (especially older minors i.e. 16 years and older) to choose independently i.e. without parental assistance, whether to participate in research’ [79].

Given well-established research into barriers to HIV prevention, which include parental influence, permission and household taboos around sexuality, it was deemed problematic to require potential participants aged 15–17 years to seek parental consent to participate in the study. For those participants who wish to involve a parent or guardian, the study is committed to facilitating this process. The Informed consent form for male participants has an optional signature line for a parent/guardian to sign, if the participant prefers this option. While national legal and ethical frameworks recommend acquiring parental consent when conducting research with minors, this study qualified for a waiver of this requirement, given the potential for harm that a young person might experience should they disclose their interest in participating in a study about sexual health to family members (given well-established taboos about discussing sexuality and sexual health in households), or the possibility that a youth may feel obstructed from participating due to this requirement, leading to unfairness.

### Protections against risk

The risk of loss of privacy will be controlled using standard data collection protocols, trained staff with regular supervision and unique participant ID numbers on all data (including specimens) rather than participant names. Research staff will take an oath of confidentiality. Psychological stress will be reduced for HIV testing that a participant may choose as a result of study intervention (though the testing itself is not a study activity) through information and education and the use of trained staff. Participants who wish to disclose their test results to key individuals in their life will be offered help and counselling to do so by clinic staff. Furthermore, a toll-free telephone/text hotline will be set up for all participants that encounter such social harms to receive support and/or advice.

### Alternative treatments and procedures

Participants from the target population invited to participate will be able to decline participation, with no consequence to them.

### Confidentiality

Confidentiality of information collected is of fundamental importance. The research team will be trained to adhere to strict confidentiality guidelines and will fully protect the confidentiality of participants possible.

### Sensitive questions

Participants do not have to answer any question that they do not want to and can stop answering the questions at any time. Additionally, skip patterns will be used to ensure to only ask more detailed questions to participants for whom these questions are relevant. Qualitative participants will not be pressured to disclose personal details they are not comfortable sharing.

### Communication with participants

No active follow-up activities are required for Aim 1; however, should individuals referred for ART initiation not be identified within one of these three data sources within 31 days of receiving their HIV positive test result, study staff will work to actively contact, trace, and link these individuals to HIV care and treatment.

For Aim 2, to ensure follow-up of participants for the purpose of data collection/verification activities, we will employ two follow-up methods that we have successfully used in previous studies:

Method #1) Participants will be asked to provide detailed personal contact information, including their phone number(s) and home addresses for themselves, a family member, or a friend/neighbour. If a participant has a personal cell phone, the cell number will be verified by calling the phone in front of the participant. Method #2) A green sticker will be discretely placed in a participant’s medical file. This green sticker will alert clinic ART nurses that the patient is a participant in our study. When a study participant returns to the clinic for routine ART refills or VL testing, clinic pharmacists or ART nurses will be asked to refer the study participant to a RC for study activities, including booster and VL result report-back messaging.

#### Retention

To observe the impact of U = U messaging on viral suppression and retention in care, no additional active follow-up activities or retention planning other than those described will be conducted. If a participant misses a clinic visit at months 6 or 12, RCs will make up to three contact attempts via phone to invite the participant to return to the clinic. If phone follow-up is not successful, research staff will work with clinic-based community tracers to contact the participant. Community tracers will not be told about the participants’ Involvement in our study.

Given that we want to observe the impact of our U = U messaging on ART adherence, viral suppression and retention in care, no additional active follow-up activities, or retention planning, other than those described, will be conducted. Should a participant be missed upon clinic attendance at months 6 or 12, RCs will make up to three attempts to make contact via phone and invite a participant to return to the clinic and complete a study questionnaire. Should follow-up by phone not be successful, research staff will work with clinic-based community tracers to contact the participant. To ensure our participants’ confidentiality, community tracers will never be told of an individual’s participation in our study. Community tracers will only know to communicate that the clinic is trying to follow up with them and to please return to the clinic.

## Dissemination

The results of the study will be analysed for publication in scientific journals and presentation at relevant scientific conferences. All presentations, abstracts, or manuscripts will be submitted to the study PIs for review and approval prior to submission and no professional writers will be used. The study’s results be communicated to key stakeholder groups, such as the South African local, provincial and the national health departments. The study’s results will also be presented to study participants, community members, and clinic staff through town hall style meetings and 1-pager flyers. Key interim results, (HIV testing, linkage, and retention in care among men), will be shared with provincial and district stakeholders on a bi-annually basis. The lessons learned from implementation will also be shared with them.

## Discussion

These trials are the first to rigorously evaluate the impact of U = U messaging on HIV testing uptake, ART initiation and achievement of viral suppression. Our intervention and trial design make three important contributions to the HIV prevention literature. First, we bring human-centered design and behavioral economics insights to the design of U = U messaging for men in South Africa. Previous research on U = U messaging has used conventional health communication and health behavior change theories to inform message development, with limited efforts to develop U = U messages that speak to men in Sub-Saharan Africa in particular. We collaborated with a human-centered design firm in South Africa, Matchboxology [31, 80], to understand how HIV and ART are perceived by men living with and/or affected by HIV, and to co-design messages that specifically resonated with men’s lived experience, aspirations, and preferences. Following our promising pilot study showing the potential of the U = U message to increase men’s uptake of HIV testing, our proposed study will be among the first to evaluate U = U messages at scale.

Second, our study examines the effect of U = U messages across the HIV care continuum: Most RCTs of behavioral interventions examine one specific behavior or outcome; for behavioral research on HIV, this is typically one step of the HIV care continuum. Our study is novel in testing whether U = U messaging can motivate MLHIV to test for HIV, start ART, adhere to ART, and achieve viral suppression (the most common rationale for U = U messages); as well as whether it can motivate men who don’t know their HIV status to seek HIV testing services. Our study will be among the first to test the effect of U = U messages across the HIV cascade in two sequential RCTs.

Third, we bring behavioral economics insights to both our intervention design and our identification of implementation targets. Building on prior work by our team, we adopt a behavioral economics lens both for the design of our U = U interventions for the two trials, and in our implementation inquiry. We apply the NUDGE framework [77, 78], an innovative behavioral diagnosis and design approach developed by members of our team, to identify specific barriers to implementation for U = U interventions based on stakeholder interviews. Our approach recognizes that providers, clinic managers, and policy makers are subject to the same biases and heuristic thinking in decision-making and in resource and attention allocation that PLHIV are in their decisions about testing, ART initiation, and retention. As a component of our preparations for the AIM 2 trial, we will evaluate the current utilization and integration of U = U messages within the clinics. This assessment aims to gauge the extent to which these messages are already in use or integrated into existing practices. Furthermore, we will regularly conduct landscape assessments to explore the nature of U = U messaging within the communities. This will involve observing and documenting the various forms of messaging and considering their potential influence on the study outcomes.

By conducting our study in two different provinces, including a province (Eastern Cape) where very little behavioral research on HIV interventions has been conducted, our study will produce generalizable knowledge about the impact of delivering theory-based U = U messaging at multiple points along the HIV care continuum for men in diverse communities with high burden of HIV. The inclusion of the Eastern Cape Province is a strength since community and health system voices from this part of the country are seldom heard. Eastern Cape’s ‘s resource-poor environment (compared to more typical study sites in Cape Town, Johannesburg or Durban) will provide a new and unique environment in which to understand implementation facilitators and barriers. We will also learn about implementation barriers that may limit the reach and adoption of successful interventions in clinical practice. If shown to be effective for South African men, our intervention will inform the implementation of HIV testing and ART adherence counselling guidelines in South Africa and globally.

## Supporting information

S1 Appendix Informed consent form. (DOCX)

## List of abbreviations

ART: Anti-retroviral therapy
BCM: Buffalo City Metro
CB-HTS: Community-based HIV testing services
EC: Eastern Cape Province
HTS: HIV testing services
MLHIV: Men living with HIV
PLHIV: People living with HIV
TasP: Treatment as prevention
U = U: Undetectable Equals Untransmittable
VL: Viral load
