# Supplementary material for: Improving HIV testing, linkage, and retention in care among South African men through U = U messaging: A study protocol for two sequential hybrid type 1 effectiveness-implementation randomized controlled trials
Source: PLoS One. 2024 Nov 25;19(11):e0309905. doi: 10.1371/journal.pone.0309905 (PMC11588259; doi:10.1371/journal.pone.0309905)
Supplement: S1 Appendix — (DOCX) [file pone.0309905.s001.docx]

**APPENDIX 1**

**Title:** **Improving HIV testing, linkage, and retention in care for men through U=U messaging**

**Sponsor:** U.S. National Institutes of Health

**Co-Principal Investigators:** Dr Andrew Medina-Marino, PhD, Desmond Tutu HIV Health Foundation (DTHF), Dr Alison Buttenheim, PhD, University of Pennsylvania, Philadelphia, USA

**Address:** Desmond Tutu HIV Centre, University of Cape Town, Faculty of Health Sciences, Anzio Road, Observatory, 7700, Cape Town

**Protocol Number:** XX (Human Research Ethics Committee, University of Cape Town)

Informed consent form: Individuals accessing HIV testing services (Aim 1)

Good morning/afternoon. My name is ____________________________ and I work for the Desmond Tutu Health Foundation (DTHF). We would like to invite you to take part in a study we are doing in collaboration with the Eastern Cape and Western Cape Departments of Health. The focus of this study is to increase men’s HIV testing. We are inviting you to participate because you are accessing HIV testing service.

I will read this consent form with you, which details the study and what will be expected of you if you agree to participate. If there is anything that you do not understand, please ask me to stop and I will explain it to you. If you have any other questions related to this study, please feel free to ask me.

PURPOSE OF THE STUDY

We are conducting this study for two main reasons:

1. We want to understand what health promotion messages are most likely to motivate men to test for HIV, and initiate treatment if they test positive for HIV, and;
2. We want to make sure that health promotion messages for men speak to the concerns and preferences of men of all ages.

STUDY PROCEDURES

If you agree to participate, you will first be asked to sign this consent form or make your thumb print mark to show that you are willing to take part in the study. If you do not want to participate, we would appreciate it if you allowed us to collect information about you and ask you a few questions in order to understand the reason(s) for your decision. This is so that we may better understand why some men may not want to participate. Should you not decide to participate or consent, you are still welcome to proceed to HIV testing. You will not be treated any differently, or provided different services just because you said no to taking part in this study.

If you agree to get involved in the study, you will be invited to complete a set of questions on a tablet computer. The survey will ask questions about your age and your health, your level of education and your employment status, your knowledge and beliefs about HIV, your home environment and community, and things that make it easy or difficult for you to access care. This should take no more than 60 minutes to complete. If you agree to participate, we will also give you a copy of this form.

After completing the questionnaire, we will direct you to a staff member for HIV counselling and testing. If you test positive, we would like your permission to follow up with you to make sure you were able to link to care for HIV treatment. Following up with you may include collecting information from your clinical or laboratory records, or contacting you in the future. For follow-up purposes, we would like to collect contact information such as a telephone number for yourself, a family member and trusted friend. If we ever need to contact someone else to try and speak with you, we will never reveal your health status or participation in our study.

RISKS, BENEFITS, AND COSTS TO YOU

Participation in this study will involve no cost to you, and you will not be paid to participate in this study. As part of this study, you will receive information about HIV and HIV treatment. Some of the questions we may ask you are personal and may make you feel embarrassed or uncomfortable. You do not need to answer these questions. If you want any further information or support related to this study, please ask me and I will do my best to answer your questions or refer you to someone who can support you. You may also become worried about people finding out about your HIV status. We promise to keep all your answers confidential and not tell anyone that you participated in our study. Your answers will provide valuable information and insights into men’s decisions to access HIV testing, care and treatment services. Furthermore, this information will help us to better support patients seeking or undergoing HIV testing, care, and treatment in the future.

CONFIDENTIALITY

Your participation in this study will be kept confidential. We will not reveal your participation, and we will make every effort to protect your privacy, confidentiality, answers, and information. You will be given a unique study number called a PIN, which will be use instead of you name or other identifiable information. Your information and answers will be stored either on a secure, password protected computer, or in a cabinet that will be kept locked at all times in our study office. Only authorised research staff will know what your special number is or be able to see your information. After five years, any personal identifying information you provide will be destroyed.

WITHDRAWAL FROM THE STUDY

Participation in this study is voluntary, you do not need to participate if you do not want to. You are free to skip any questions you do not want to answer. You can also choose to stop the questionnaire and withdraw your participation at any time. Your HIV testing, or any care and treatment services you may want will NOT be affected if you choose to not take part in this study or withdraw after you consent.

PROBLEMS OR QUESTIONS

If you ever have any questions or experience harm because of the study, please contact the Project Manager, Nkosiyapha Sibanda at 087 821 1109 or [Nkosiypha.Sibanda@hiv-research.org.za](mailto:Nkosiypha.Sibanda@hiv-research.org.za) . This research has been approved by the University of Cape Town Human Research Ethics Committee (an independent committee established to help protect the rights of research participants). If you have any concerns about the way the study is being carried out, you may contact the committee chair on 021 650 1236 or [marc.blockman@uct.ac.za](mailto:marc.blockman@uct.ac.za).

DECLARATION TO PARTICIPATE

By signing below, I _____________________________consent voluntarily to participate in the study entitled: *“***Improving HIV testing, linkage, and retention in care for men through U=U messaging”**

**I declare that:**

1. I have read this consent form or had it read to me.
2. I understand the aims of the research and have had an opportunity to ask questions.
3. I understand what my involvement in the study will entail and agree to participate.
4. I understand that I do not have to give any information that I am not comfortable sharing.
5. I understand that I can withdraw from the study at any point if I feel the need to.
6. By signing this form, I do not give up any rights that I have as a study participant.

**If participant consents:**

**Name of Participant:** ___________________________ **Signature of Participant:** _________________________

**Date** (dd/mm/yyyy): _____________________________

***If illiterate (witness to sign)*: I have witnessed the accurate reading of the consent, and the participant has had the opportunity to ask questions. I confirm that the participant has given consent freely.**

**THUMB PRINT**

Print name of witness: __________________________

Signature of witness: ___________________________

Date (dd/mm/yyyy): ____________________________

**DECLARATION BY RESEARCH ASSISTANT**

I *(RA name)* _______________________________ declare that:

- I explained the information in this document to ______________________________ *(name of participant)*
- I encouraged him/her to ask questions and took adequate time to answer them
- I am satisfied that he/she adequately understands all aspects of the research, as discussed above
- I did not use an interpreter

| Y | N |
| --- | --- |

A copy of this Informed Consent Form has been provided to the participant

Signed at (*place*) _________________________ on (*date- dd/mm/yyyy*) _______________

Signature of research assistant _________________________
